# Unearthing FLVCR1a: tracing the path to a vital cellular transporter

**DOI:** 10.1007/s00018-024-05205-3

**Published:** 2024-04-06

**Authors:** Veronica Fiorito, Emanuela Tolosano

**Affiliations:** Molecular Biotechnology Center (MBC) “Guido Tarone”, Department of Molecular Biotechnology and Health Sciences, University of Torino, Via Nizza 52, 10126 Turin, Italy

**Keywords:** FLVCR1, FLVCR1a, Heme, Choline

## Abstract

The Feline Leukemia Virus Subgroup C Receptor 1a (FLVCR1a) is a member of the SLC49 Major Facilitator Superfamily of transporters. Initially recognized as the receptor for the retrovirus responsible of pure red cell aplasia in cats, nearly two decades since its discovery, FLVCR1a remains a puzzling transporter, with ongoing discussions regarding what it transports and how its expression is regulated. Nonetheless, despite this, the substantial body of evidence accumulated over the years has provided insights into several critical processes in which this transporter plays a complex role, and the health implications stemming from its malfunction. The present review intends to offer a comprehensive overview and a critical analysis of the existing literature on FLVCR1a, with the goal of emphasising the vital importance of this transporter for the organism and elucidating the interconnections among the various functions attributed to this transporter.

## Introduction

Accumulating studies, published since the late 90 s, contributed to bring to the fore the receptor isoform 1a for the Feline Leukemia Virus Subgroup C (FLVCR1a): initially identified as the receptor for the retrovirus responsible of red cell aplasia in cats, a seminal work published on Cell in 2004 [1] demonstrated its role as a human heme exporter. After that, several papers highlighted the essential role of FLVCR1a in erythropoiesis [2–6], the process with the highest rate of heme synthesis, and subsequent studies dissected FLVCR1a function in different tissues [7–9, 10]. Furthermore, specific disorders resulting from mutations in *FLVCR1* gene have been documented, and FLVCR1a dysregulation has been associated with various pathological conditions, including cancer [11]. Collectively, these findings have sparked increased interest in this protein and underscored its potential significance in therapeutic interventions. Remarkably, the necessity for a comprehensive characterization of this transporter has become increasingly urgent in recent months, following the publication of new studies that have cast doubt on the existing literature. Indeed, emerging research [12, 13, 14] has raised queries regarding FLVCR1a's established role as a heme exporter and has put forth an alternative function as a choline importer, proposing a re-evaluation of prior findings. These studies have stimulated an ongoing debate, which remains unresolved as of the writing of this manuscript.

By critically analysing the available literature on FLVCR1a, the following paragraphs aim to guide the reader toward a thorough and unbiased summary of the discoveries that have surfaced over approximately two decades of research on this transporter. We aspire that this review will foster a more informed and discerning perspective on the literature concerning FLVCR1a, while also deepening the comprehension of the protein's pathophysiological significance.

## The discovery of FLVCR1a

In the late 90 s two different research groups, based one in the United Kingdom (the group of Kabat et al. [15]) and the other in the USA (the group of Abkowitz et al. [16]), got interested in a peculiar receptor exploited by the FeLV-C retrovirus, a mutant of FeLV-A retrovirus, to infect the host cells. After its successful cloning, the receptor was named FLVCR. Subsequently, the name was changed in FLVCR1, to distinguish it from a similar receptor (FLVCR2) sharing high sequence identity with FLVCR1 [17]. Finally, the name FLVCR1a was adopted when it was discovered that the *FLVCR1* gene (that in human was mapped to chromosome 1q31.3) also codify for another shorter isoform, *FLVCR1b* [18].

Feline leukemia viruses (FeLVs) are a group of retroviruses causing prevalent contagious infections of domestic cats that result in proliferative, degenerative, and immunosuppressive disorders. Particularly, FeLV-C infection has been tightly associated with the development of a fatal pure erythrocyte aplasia, which involves depletion of erythroid colony forming units (CFU-E) and burst-forming units (BFU-E). FVCR1a is highly specific for FeLV-C, as it does not confer susceptibility to infections of FeLV-A, FeLV-B, amphotropic murine leukemia viruses, or RD114 feline endogenous retrovirus. The binding specificity of FeLV-C to its receptor relies on a discrete 30 aminoacid-long variable region (vr1) of the glycoprotein (the SU protein) present on its envelope. This small portion acts as a dominant negative factor, potentially impeding the activity or surface presentation of FLVCR1a, and it is determinant for the development of aplastic anemia in the cat. This observation led to the conclusion that the development of red cell aplasia was not related to the FeLV-C virus per se, but to the interaction of the virus with its specific receptor, likely altering a function mediated by the receptor itself. Remarkably, except for the naturally resistant cell lines NIH 3T3 (murine fibroblast) and NRK 52E (rat kidney epithelium), the FeLV-C virus can infect several cell types derived from different organisms, but in cats the aplasia is restricted to red cells, indicating that erythroblasts may be either exceptionally sensitive to secondary sequelae of infection or critically dependent on the normal function of FLVCR1a, or that this receptor has a nonessential/redundant function in other cells [15, 16].

## Structural insights and putative operational mode

As mentioned above, FLVCR1a is expressed not only in cats’ cells, but in different organisms and cell lines. Base pairs in the coding regions of feline and human cDNAs share an 88% identity. Moreover, feline FLVCR1a exhibits an 83% amino acid identity and an 89% similarity to the human FLVCR1a protein [16].

FLVCR1a protein is about 560 aminoacids long and it has a molecular mass of about 55–70 kDa (depending on glycosylation [17]). The protein is composed by 12 hydrophobic membrane-spanning domains and 6 hydrophilic extracellular loops, with the amino and carboxyl termini in the cytosol. Moreover, it shows three N-glycosylation sites, two of which in the third extracellular domain and one in the transmembrane domain 11 [17]. Putative sites of protein kinase C phosphorylation, casein kinase II phosphorylation, and N-myristoylation were also identified. The receptor region that are critical for viral infection are the extracellular loops 1 and 6 [17].

Based on its characteristics and membrane topology, FLVCR1 protein is considered a member of the SLC49 Major Facilitator Superfamily (MFS) of transporters, a large ancient superfamily including at least 17 evolutionarily distinct branches, each specialized in the transport of a particular category of solutes [19]. The MFS members are present in all domains of life and often exist in multiple gene copies. Among MFS proteins, the most studied are glucose transporters (GLUTs).

MFS transporters can be categorized into three primary clusters, based on the mode of transportation: uniporters convey a solitary substrate; symporters transport a substrate alongside a coupled ion (typically protons); and antiporters transport a substrate and a co-substrate in opposing directions, with one's binding dependent on the prior release of the other. Uniporters do not necessitate external energy input, but generally, they can only move substrates in the direction of their concentration gradient. In contrast, symporters and antiporters can harness the energy stored in the concentration gradient of their coupled ion or co-substrate to transport substrates against their concentration gradient. FLVCR1a appears to be closely related to organic anion antiporters or symporters, although so far the precise mechanism of FLVCR1a-mediated substrate transport and the coupled anion have not been identified.

As FLVCR1a belongs to the MFS of transporters, it can be predicted that it shares the same structure and operating mode of the other family members. Indeed, although extremely heterogenous in substrate specificity, the MFS members display the same structural fold.

The twelve transmembrane helices (TM1–TM12) are organized into two structurally similar domains, the N‑domain (TM1–TM6) and the C‑domain (TM7–TM12). The substrate-binding site is located in the centre of the protein and is composed of residues from both the N‑domain and the C‑domain. Regarding the operational mode, the current model is a two-step, ‘clamp-and-switch’ mechanism. This means that, during the transport of its carrier, an MFS transporter progresses through a conformational cycle that involves at least four conformational states and that, in case of a cargo transported inside the cell from the extracellular space, can be summarized as follows: the outward open state (with an access to the substrate binding site facing the extracellular side, to allow substrate binding), the outward-facing occluded state (with the access facing the extracellular side closed by a “gate”, a set of specific residues); next, the N-domain and the C-domain complete a rocker-switch-type rotation on their axis, resulting in the exposure of the binding site toward the cytosol (inward open state). Finally, an inward-facing occluded state (with the intracellular access closed by a “gate”) precedes the return to the outward open state to start again the cycle [20]. No energy input, other than the energy stored in the concentration gradient of the substrate, is required to drive the conformational cycle, and it seems that also in proton-coupled antiporters and symporters the protons do not provide the energy input, being their function limited to enable substrate binding [20].

At the time of writing, different works on FLVCR1a structure have appeared [21, 22], although not already peer-reviewed, that seem to confirm at least in part the predicted structure described.

## FLVCR1a cargos: an ongoing debate

In 2004 Quigley et al. [1] clarified, by a series of experiments exploiting both ^55^Fe-heme and the heme analog zinc-mesoporphyrin (ZnMP), that FLVCR1a transports heme and, specifically, that FLVCR1a is a heme exporter. This discovery was a crucial step in the field, as several reports, beginning in the 1950s, hinted at the existence of a heme-binding protein on the outer layer of mammalian cells, including enterocytes, hepatocytes, and hematopoietic cell lines [23, 24, 25] but the identity of this protein was unknown. Moreover, the identification of a heme exporter on cell surface implied, for the first time, that cells could exchange heme with each other and not just iron. In washout experiments, the authors showed that NRK 52E cells pre-loaded with ^55^Fe-heme retained a higher quantity of heme than FLVCR1a over-expressing NRK 52E cells. The same results were obtained with ZnMP, a heme analog that also acts as an inhibitor of the microsomal heme-degrading enzymes heme oxygenases, helping to exclude confounding contribution of heme degrading processes in the analysis [1, 26]. The experiments also proved that heme (and ZnMP) is exported by FLVCR1a as an intact molecule across the cell surface membrane. Our laboratory also validated an enhanced retention of ZnMP in FLVCR1a-deficient *vs* proficient cells in washout experiments using Caco2 cells, a human colorectal cancer cell line [27].

In another study by Abkowitz’s group [26], additional experiments demonstrated that FLVCR1a-mediated heme export is completely dependent on the extracellular presence of a heme-binding protein, with hemopexin being extremely more efficient in promoting heme export as compared to albumin, another heme binding protein but with lower affinity. Indirectly consistent with this, we observed that fibroblasts and lymphoblastoid cell lines derived from patients with hereditary sensory and autonomic neuropathies (HSANs), who harbor mutations resulting in reduced FLVCR1a functionality, exhibit increased heme accumulation and cell death upon heme synthesis induction, and survival is rescued by the addition of hemopexin to the culture medium [28].

In vitro experiments by Abkowitz’s group have also showed that FLVCR1a is not involved in unconjugated bilirubin transport, whereas, in addition to heme and ZnMP, it can also export non-metallo planar porphyrins, as coproporphyrin and protoporphyrin IX [26]. Nevertheless, the physiological relevance of this function is uncertain, and (ATP)-binding cassette efflux transporter G2 (ABCG2) is still considered a major exporter for porphyrins [29, 30].

In vitro experiments also showed that FLVCR1a can export both endogenously synthesized heme and heme provided exogenously [26]. However, also in this case the physiological relevance of FLVCR1a export of externally supplied heme has been debated, as most literature studies on this topic, including our own in vivo experiments in liver specific *Flvcr1a*-null mice injected with hemin [7], typically involve the administration of very high heme doses, which greatly exceed the concentrations usually encountered by cells in physiological conditions. Corroborating this theory, in the same liver-specific *Flvcr1a*-null mice mentioned above, we observed that, upon treatment with the hemolytic agent phenylhydrazine, *Flvcr1a*-null mice behaved as the wild-type counterpart, suggesting that FLVCR1a is dispensable for the response to exogenous heme released from damaged red blood cells [7]. Likewise, animals affected by sickle cell anemia exhibit reduced levels of *Flvcr1a* expression and an induction of heme oxygenases [31], strengthening the concept that exogenous heme has to be degraded rather than exported. Furthermore, we observed duodenal heme oxygenase 1 induction in mice maintained on a heme-supplemented diet, whereas *Flvcr1a* expression was unaffected [9]. Taken together, these findings cast doubt on the notion of FLVCR1a playing a role in the export of externally provided heme. Conversely, a multitude of investigations conducted in various cellular and animal models agree with a role of FLVCR1a in the management of endogenously synthesized heme. Indeed, in vitro experiments demonstrated that, following stimulation of heme synthesis with the heme precursor 5-aminolevulinic acid (ALA), FLVCR1a-downmodulated HUVECs [8], SHSY-5Y cells [27], HeLa cells [18] and Caco2 cells [9], as well as primary hepatocytes derived from *Flvcr1a*-knockout livers [7] and fibroblasts or lymphoblastoid cell lines (LCLs) derived from patients carrying *FLVCR1* mutations[28, 32], all accumulated more heme. More importantly, ALA-induced heme accumulation was prevented by the concomitant administration of the heme synthesis inhibitor succynilacetone (SA) [7, 9], confirming the intracellular biosynthetic origin of heme deposits in these cells. In line with these in vitro observations, also ALA administration in vivo in liver-specific *Flvcr1a*-null mice resulted in hepatic heme accumulation [7]. Noteworthy, the accumulation of endogenously synthesized heme within cells lacking *Flvcr1a* was observed even without any manipulation of heme synthesis or degradation: using high-performance liquid chromatography measurements, a notably higher quantity of heme was detected in the cell pellet retrieved from NRK 52E cells, which do not express FLVCR1a, in contrast to FLVCR1a-overexpressing NRK 52E cells. Conversely, heme was undetectable in the culture supernatants from NRK 52E cells, whereas a significant amount of heme was detected in the supernatant of FLVCR1a-overexpressing NRK 52E cells [26]. On the same line, erythroblasts from *Flvcr1*-knockout mice displayed elevated heme levels across all erythropoietic stages in comparison to those found in control mice [4]. However, it is crucial to recognize that in alternative models of *Flvcr1a* downregulation, steady state heme levels (without the acute induction of heme biosynthesis) do not consistently exceed those in controls. This inconsistency likely indicates the activation of compensatory mechanisms aimed at preserving overall heme homeostasis, which may involve the reduction of heme biosynthesis. In line with this, the connection between FLVCR1a and heme synthesis was further sustained by the detection of reduced ALAS1 expression and/or activity in various cell types (including HUVECs, Caco2 cells, C80 cells, SKCO1 cells) upon FLVCR1a downregulation [8, 27, 33], whereas FLVCR1a overexpression in Caco2 cells resulted in enhanced ALAS1 levels [27]. Similarly, lymphoblastoid cell lines derived from HSANs patients, harboring defective FLVCR1a function, exhibited decreased expression of ALAS1 [28]. The widely recognized feedback inhibition of ALAS1 by heme operates at various levels, including transcriptional and post-translational regulation. Thus, the influence of FLVCR1a expression on ALAS1 activity not only helped establish a link between FLVCR1a and endogenous heme synthesis, but also contributed to support the idea that FLVCR1a facilitates the export of internally synthesized heme, acting as a significant player in the mechanism of heme-mediated feedback inhibition of ALAS1. Finally, FLVCR1a was demonstrated to be essential for maintaining a steady supply of newly synthesized heme to support the proper functioning of hemoproteins [7], further supporting the link between heme synthesis and FLVCR1a.

Therefore, collectively, the substantial body of data accumulated over the past two decades supports the notion of FLVCR1a having a role in exporting endogenously synthetized heme. However, it's important to acknowledge that these studies come with both strengths and limitations. On one hand, the strengths of these investigations stem from their appropriateness in terms of experimental settings: they take into account the physiological expression patterns of FLVCR1a, provide cells with the capacity to modulate its expression, and maintain an environment conducive to its proper function. This includes the presence of heme binding proteins and the supply of its potential substrates from a topologically correct compartment. On the other hand, there are notable limitations: these studies do not include real-time tracking of heme transport by FLVCR1a, and a solved structure of the protein that would definitively confirm its ability to transport heme. Consequently, the experiments cannot entirely rule out the possibility that the effects observed on heme accumulation and heme metabolism following FLVCR1a dysregulation may be secondary consequences arising from alterations in other heme transporters or heme metabolic proteins that are indirectly influenced by FLVCR1a function.

**Fig. 1:**
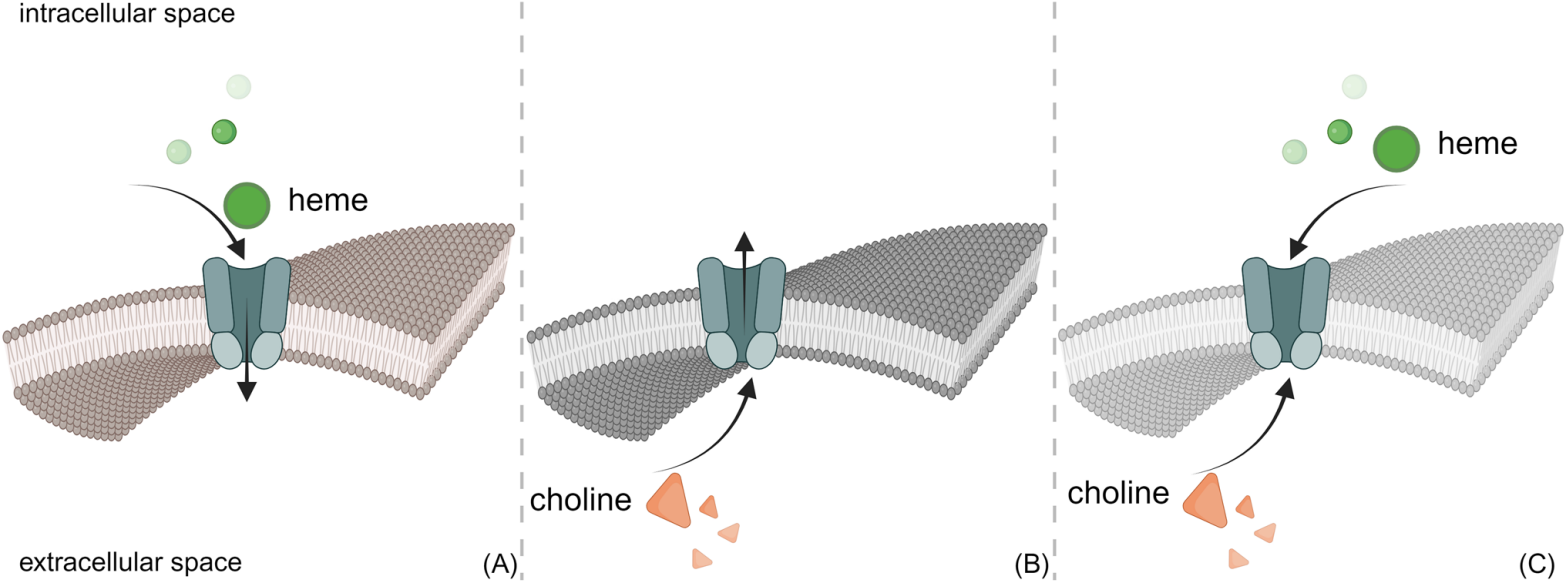
FLVCR1a putative cargos. Several studies, taking into account the physiological expression patterns of FLVCR1a, enabling cells to modulate its expression and maintaining an environment conducive to its proper function, suggest that FLVCR1a functions as a heme exporter. However, these studies lack real-time tracking of heme transport by FLVCR1a and a solved protein structure that would definitively confirm its ability to transport heme. On the contrary, other studies, which include the tracing of labeled choline and the detection of choline-related metabolites, clearly indicate that FLVCR1a is involved in choline import. Nevertheless, most of these studies did not analyse cells with their natural level of FLVCR1a expression and the ability to adjust its expression. Additional research is required to elucidate whether FLVCR1a primarily serves as a heme exporter (**A**), a choline importer (**B**), or potentially as a dual-purpose transporter (**C**). The figure was created with BioRender.com

Further adding complexity to the issue, recent publications have indicated that FLVCR1a may serve not only as a heme exporter but also, or alternatively, as a choline importer, suggesting dual or alternative functional roles for this protein (Fig. 1).

Two studies published in 2022 [34, 35] identified a possible connection between choline metabolism and FLVCR1a. In line with these observations, in 2023 Kıvanç Birsoy’s group [12] identified FLVCR1 as a gene whose variants affect choline serum levels in humans, and observed that FLVCR1a-knockout Hek293T and HeLa cells exhibited reduced levels of choline, betaine and phosphocholine, as well as phosphatidylcholine species, and reduced incorporation of isotope-labelled choline in its downstream metabolites (phosphocholine, betaine and glycerophosphocholine) as compared to FLVCR1a-knockout cells complemented with a cDNA expressing FLVCR1a. Moreover, they reported that complementation of knockout cells with *FLVCR1a*, but not with the *FLVCR1b* isoform, rescued the phosphocholine levels. Similarly, the authors found significant drops in choline, betaine and phosphocholine levels in *Flvcr1* knockout embryos when compared with controls. As the expression/activity of choline kinase alpha (the enzyme responsible for choline trapping into cells) and of enzymes involved in phospholipids synthesis were unaffected by FLVCR1a loss, the authors explored the hypothesis that FLVCR1a might serve as the long-sought high-affinity choline transporter in mammalian cells, being the expression of the high-affinity choline transporter SLC5A7/CHT1 predominantly restricted to cholinergic neurons. Taken advantage of radiolabeled choline ([Methyl-3H]Choline) uptake assays, they demonstrated that the loss of FLVCR1a blocked choline uptake in HEK293T and HeLa cells in a dose- and time-dependent manner, and that complementation of knockout cells with *FLVCR1a* rescued the uptake of choline. Their findings led to the conclusion that FLVCR1a functions as a choline importer. Moreover, they claimed that this role is vital for cell proliferation, as the absence of FLVCR1a results in reduced cell growth. Notably, these effects were observed exclusively in cells cultured on choline limitation. In contrast, when choline was reintroduced in the culture medium, FLVCR1a was dispensable, suggesting the activation of compensatory mechanisms. Among them, the authors demonstrated that the survival of *FLVCR1a*-deficient cells depends on (1) the expression of FLVCR2, that they show also to transport choline, (2) the activation of various phospholipid synthetic genes, (3) the promotion of cholesterol synthesis, and (4) the activation of the lysosomal phospholipid salvage pathway, consisting in the SPNS1-mediated transport of lysophosphatidylcholine, a lysosomal breakdown product of extracellular lipids, into the cytosol, which provides a source for choline [12]. These compensatory systems which, as mentioned earlier, are inadequate in conditions of choline limitation but are essential during choline repletion, become unnecessary when cells are grown in the presence of supraphysiological choline.

Some of these findings align with our observations in endothelial cells [36]. Specifically, we found that FLVCR1a downmodulation in Sk-Hep1 cells, a widely acknowledged model for liver sinusoidal endothelial cells, and in breast-tumor-derived endothelial cells, both cultured in the presence of choline, enhances cholesterol synthesis. Conversely, FLVCR1a overexpression induces the opposite phenotype. In our study, we observed that the increased cholesterol production was linked to an enhanced reductive carboxylation of glutamine, a pathway typically promoted to support de novo lipogenesis. This resulted in heightened production of mitochondrial citrate, a crucial substrate for cholesterol synthesis upon export to the cytosol. Consequently, inhibition of the mitochondrial citrate carrier, involved in citrate export toward the cytosol, normalized cholesterol synthesis in *FLVCR1a*-silenced cells. Furthermore, we were able to ascribe this metabolic adaptation to the decreased heme biosynthesis. Specifically, ALAS1 activity was found to be reduced in *FLVCR1a*-silenced cells and increased in *FLVCR1a*-overexpressing cells. Additionally, restoring ALAS1 activity levels in *FLVCR1a*-overexpressing cells was sufficient to rescue cholesterol synthesis. Therefore, this research suggests that the perturbation in cholesterol synthesis due to FLVCR1a alteration relies on ALAS1. Hence, a tentative synthesis of our findings with the results from Birsoy’s group suggests that the increased cholesterol biosynthesis observed in *FLVCR1a*-silenced cells may serve to compensate for choline homeostasis defects, and that both administration of supraphysiological choline and normalization of ALAS1 activity can mitigate this requirement. This suggests that if impairment of choline import promotes cholesterol synthesis, it likely does so by modulating heme biosynthesis. Nevertheless, alternative interpretations could explain these data, and the design of appropriate experiments that encompass analyses of both heme and choline metabolism is required to effectively address this issue.

Another controversial aspect is the observation reported in Birsoy’s work [12] about the proliferation rate of *FLVCR1a*-deficient cells, which are not in agreement with our own observations, as well as with that of other groups, in several tumor and non-tumor cell lines. Indeed, we reported significant decrease of cell proliferation of *FLVCR1a*-silenced cells in choline repleted conditions both in vitro and in vivo [1, 8, 27, 33, 37–39]. Moreover, it's important to note that most of the experiments of Birsoy’s group were conducted by comparing cells lacking FLVCR1a (knockout) with cells overexpressing FLVCR1a (the complemented cells). They did not analyze cells with their natural level of FLVCR1a expression and the ability to adjust its expression. Nevertheless, despite some limitations, the study [12] undoubtedly unveiled a connection between choline and FLVCR1a and the authors proposed FLVCR1a, and its paralog FLVCR2, as the major transporters of choline in mammals.

In agreement with this paper, a parallel work by Itaru Hamachi’s group identified FLVCR1 as a critical gene for human phosphatidylcholine metabolism [13]. They developed a strategy to convert organelle lipid phenotypes into a simple fluorescence readout for genome-wide screening, named organelle-selective click chemistry coupled with flow cytometry (O-ClickFC), based on the simultaneously tagging of phosphatidylcholine in various organelle membranes with different-colored fluorescent dyes. Using this technique, they were able to identify genes involved in phosphatidylcholine metabolism and trafficking. Among them, they identified *FLVCR1* as a gene whose deficiency strongly reduces phosphatidylcholine labeling in various cell lines (K562, A549 and HeLa cells) and decreases K562 cells total choline levels as well as proliferation. Moreover, they reported that complementation of *FLVCR1a*-knockout cells with FLVCR1a rescued the phosphatidylcholine labeling.

Again, the analyses were conducted by comparing cells lacking FLVCR1a (knockout) with cells overexpressing FLVCR1a (the complemented cells). Moreover, in our opinion, the experiments did not provide a clear resolution regarding whether the observed choline and phosphatidylcholine abnormalities in FLVCR1a-knockout cells are a result of heme metabolism dysregulation or, as suggested by the authors, are directly related to the choline-importing function ascribed to FLVCR1a. However, regarding this point, as previously mentioned, at the time of writing this review, several studies on FLVCR1a and choline have appeared [21, 22, 14]. Although most of them have not yet undergone peer review, these studies provide additional evidence confirming the choline transport capability of FLVCR1a.

Finally, Itaru Hamachi’s group also observed that heme or its derivatives do not interfere with the azido-choline (N3-Cho) tracer uptake in K562 cells, suggesting that choline import is not hindered by heme or its derivatives [13]. Also in this case, it's important to note that, in our view, this experiment does not fully address the potential reciprocal competition between heme export and choline import mediated by FLVCR1a. This is because the heme derivatives used in the experiment were supplied in the extracellular environment. To more accurately reflect the real situation faced by plasma membrane FLVCR1a, it would be necessary to investigate the effects of intracellular heme derivatives on choline uptake, which has not been tested in this study.

Consistent with the findings from Birsoy’s group and Hamachi’s group, and aligning with their conclusions, a recent study [14] by Long N. Nguyen’s group further affirms that FLVCR1a functions as a choline transporter. In their investigation, the authors demonstrated a reduction in choline import in A549 cells with downregulated FLVCR1a, coupled with a slight decrease in phosphatidylcholine and sphingomyelin levels. Moreover, they established the conservation of the choline-importing function of FLVCR1a across various species (fly, fish, frog, chicken, and mouse) and highlighted its independence from a cation gradient. Additionally, the study revealed that FLVCR1a facilitates choline uptake downhill its concentration gradient. This implies that the intracellular choline levels, along with its utilization by intracellular choline-consuming enzymes, serve as a limiting factor influencing the rate of choline influx mediated by FLVCR1a. Overall, this research more convincingly supports a physiological involvement of FLVCR1a in choline transportation. Nevertheless, even though the authors rule out FLVCR1a-mediated transport of substances such as L-carnitine, acetylcholine, betaine, and serotonin, they demonstrate that it also facilitates the transport of ethanolamine. This sustains the possibility that FLVCR1a may transport multiple substrates, not limited exclusively to choline.

Summarizing, both the experiments assessing FLVCR1a's abilities in exporting heme and importing choline show strengths and weaknesses and, as of now, a comprehensive understanding of the substances transported by FLVCR1a remains elusive. More work is required to fully clarify what cargo FLVCR1a transports and the significance of this transportation in physiological processes. This involves several necessary steps, such as publishing the structure of FLVCR1a and conducting experiments under specific conditions. These conditions include: (1) ensuring the presence of heme binding proteins in the environment, (2) properly preparing heme (which is a challenging molecule to handle due to its sticky nature), (3) using physiological concentrations of the cargo, (4) supplying the cargo from the correct topological compartment, and (5) validating the physiological importance of the results in suitable cellular and animal models that express FLVCR1a at physiologically relevant and adjustable levels. These experiments necessitate the skills and knowledge of various experts from different professional backgrounds, as well as effective collaboration among the diverse research groups engaged in these two distinct lines of investigation.

## Regulation of *FLVCR1* gene expression

Much like the ambiguities surrounding the substances transported by FLVCR1a, nearly two decades since its original identification, the control of FLVCR1a expression remains largely elusive.

In 2004, Abkowitz’s group reported that a region of the *FLVCR1* promoter contains four potential STAT5a binding sites, as well as consensus GATA-1, GATA-2, c-myb and NF-E2 binding sites, providing potential mechanisms for the upregulation of *FLVCR1* transcription during early erythroid commitment and differentiation [1]. Subsequently, in 2006, a study conducted by E. Nečas research group examined the impact of lipopolysaccharide (LPS) and bleeding on the expression of intestinal proteins. Their findings revealed a reduction in mouse intestinal *Flvcr1* mRNA levels upon LPS treatment [40], suggesting a potential regulation of *Flvcr1* transcription by factors associated with the inflammatory response. Finally, in 2014, our laboratory revealed that *Flvcr1* gene expression is modulated in different tissues and cell lines in response to hypoxia [41]. Chromatin immunoprecipitation analyses demonstrated that the hypoxia inducible factor 2α (HIF2α) and the HIF-dependent transcription factor ETS1 (v-ets avian erythroblastosis virus E26 oncogene homolog 1) bind at the region − 318/ + 39 of the *Flvcr1* promoter. Analyses in Caco2 cells in which HIF2α or ETS1 were silenced or overexpressed demonstrated that, both HIF2α and ETS1 are involved in the transcriptional regulation of *Flvcr1a* and that HIF2α is absolutely required for *Flvcr1a* induction upon hypoxia [41].

## The role of FLVCR1a in cell differentiation

Despite a restricted knowledge on the regulatory sequences within the *FLVCR1* gene dictating its expression, several studies have contributed to elucidate the conditions that either encourage, or are influenced by, changes in FLVCR1a expression.

The human erythroleukemia cell line HEL is capable of spontaneous and induced globin synthesis [42] and, together with K562 cells [43], have been exploited by Abkowitz’s group to analyze *FLVCR1* expression [1]. Interestingly, they observed that *FLVCR1* mRNA and protein levels were high in mobilized peripheral blood CD34^+^ stem/progenitor cells and in hematopoietic cell lines with erythroid features (K562, HEL-DR), but cell surface protein expression was absent in a more mature erythroid cell line, HEL-R [44], with spontaneous hemoglobinization, which derives from HEL-DR [1]. In line with this, CFU-E derived from mobilized human PB CD34^+^ stem/progenitor cells showed a higher expression of *FLVCR1* as compared to other hematopoietic progenitor cells [1]. In addition, the inhibition of *FLVCR1* cell surface expression or function significantly impaired the ability of K562 cells to undergo erythroid differentiation [1]. Collectively, these data indicated that *FLVCR1* expression decreases as CFU-E differentiate and hemoglobinization proceeds. To gain a more profound understanding of the role of this transporter in the erythropoietic system, in 2008 Abkowitz's group generated constitutive (*Flvcr1*^+/–^) and inducible bone marrow-specific (*Flvcr1*^+/flox^;Mx-cre) *Flvcr1* mutant mice [2]. Interbred *Flvcr1*^+/–^ animals yielded no null offspring (*Flvcr1*^–/–^), with intrauterine deaths occurring before embryonic day 7.5 or between E14.5 and E16.5, underlying the vital role played by *Flvcr1*. Mutants had abnormal limb, hand, and digit maturation, flattened faces, hypertelorism, and pale liver, whereas cardiac, pulmonary, and genitourinary systems appeared normal. Moreover, they showed a block in erythroid maturation at the proerythroblast stage. Postnatal mice lacking *Flvcr1* in the bone marrow (*Flvcr1*^flox/flox^;Mx-cre) showed pale paws, cardiomegaly and splenomegaly. In addition, they exhibited pronounced iron loading in hepatocytes, duodenal enterocytes and splenic macrophages, associated to high liver hepcidin levels. These mice developed a severe hyperchromic macrocytic anemia, reticulocytopenia and a block in erythroid maturation at the proerythroblast stage. In line with this, erythroid colony assays showed absence of CFUs-E and suboptimal expansion of BFUs-E. Conversely, mouse recipients transplanted with bone marrow cells overexpressing *Flvcr1* displayed mild hypochromic, microcytic anemia [2]. The authors were also able to demonstrate that the erythroid defect observed in animals deficient for FLVCR1 was due to the lack of *Flvcr1* in hematopoietic cells, and not in the microenvironment [2]. Collectively, these findings highlighted the essential role of FLVCR1 in the differentiation of erythroid cells. Nevertheless, the two mouse models generated by Abkowitz’s group presumably lacks both the *Flvcr1a* and *Flvcr1b* isoforms encoded by the *Flvcr1* gene, as the inactivation of the gene was achieved by deleting the third exon, which is shared by both isoforms.

A more detailed understanding of the phenotypic outcomes resulting from the deletion of the sole *Flvcr1a* isoform emerged through the analysis of the constitutive [18] and the inducible [5] bone marrow specific *Flvcr1a*-knockout models (*Flvcr1a*^−/−^ mice and *Flvcr1a*^flox/flox^; Mx-Cre mice, respectively) generated in our lab. The phenotype observed in postnatal mice lacking *Flvcr1a* in the bone marrow (*Flvcr1a*^flox/flox^; Mx-Cre) [5] closely resembled that of mice lacking both FLVCR1 isoforms. Conversely, in the case of constitutive *Flvcr1a*-specific knockout embryos (*Flvcr1a*^*−*/−^) [18], there were discernible distinctions when compared to the corresponding *Flvcr1*^*−*/−^ model developed by Abkowitz's group. *Flvcr1a*^−/−^ embryos died between E14.5 and birth and although they exhibited skeletal abnormalities [18], which resembled the ones observed in mice lacking both *Flvcr1* isoforms, the erythroid phenotype of these embryos was less pronounced. Indeed, an initial study suggested normal erythroid differentiation [18], and only subsequent more in-depth analyses of their BFU-E and CFU-E revealed impaired colony formation in these embryos [5]. The inconclusive nature of these studies regarding the role of FLVCR1a in erythropoiesis prompted Abkowitz’s group to conduct additional experiments. To investigate the specific roles of FLVCR1a and FLVCR1b in erythropoiesis, they reconstituted the bone marrow of *Flvcr1a*^flox/flox^;Mx-Cre mice with *Flvcr1*-deleted marrow cells transduced with a retroviral vector encoding either FLVCR1a or FLVCR1b. Mice transplanted with marrow cells expressing FLVCR1a remained alive and healthy, maintaining normal hematocrits and hemoglobin levels for at least 6 months after transplantation. In contrast, mice transplanted with marrow cells expressing FLVCR1b succumbed to severe anemia, highlighting that FLVCR1a, rather than mitochondrial heme export by FLVCR1b, is capable of supporting erythropoiesis [4].

Despite these works contributed to clarify the importance of FLVCR1a in erythropoiesis, further revelations introduced additional layers of complexity to the matter. Indeed, many of these insights were predominantly gleaned from in vitro explorations utilizing murine and human cells, along with in vivo examinations employing mouse models. Nevertheless, the matter was also investigated through the examination of zebrafish [5, 39]. During zebrafish development *Flvcr1a* transcript was detectable as a maternal mRNA in unfertilized eggs and then increased from 8 to 96 h post fertilization (hpf) [5]. The *Flvcr1b* transcript was detectable by 24 hpf and increased throughout development [5]. Morphants lacking both Flvcr1 isoforms displayed a delay in development by 24 hpf, and had a shorter body than controls, a ventrally bent tail and smaller heads by 48 hpf. More than 90% of them developed hydrocephalus and lacked yolk extension and had fewer circulating erythroid cells. The introduction of *Flvcr1a* cRNA into these morphants restored the count of circulating erythroid cells, but it did not influence heme content or globin expression. In contrast, the injection of *Flvcr1b* cRNA had a minimal impact. Notably, when *Flvcr1a* and *Flvcr1b* cRNAs were concurrently injected into these morphants, the erythroid defect was completely restored [5]. These discoveries, coupled with additional experiments, led to the conclusion that in zebrafish, FLVCR1a plays a crucial role in promoting the expansion of erythroid progenitors, but is insufficient to drive their final maturation [5]. On the other hand, FLVCR1b on its own cannot sustain expansion, but is essential for differentiation [5].

In summary, the contribution and essentiality of FLVCR1a and FLVCR1b in specific processes supporting erythropoiesis remain controversial. Nevertheless, despite some discrepancies arising from variations in animal species (mouse *versus* zebrafish) and the types of erythropoiesis (fetal *versus* adult) under investigation, these studies collectively endorse the notion that maintaining an appropriate expression of *Flvcr1* gene is crucial for the proper progression of erythroid cell expansion and differentiation.

Moreover, a common concept arising from these studies is the reduction of FLVCR1a levels during erythroid differentiation. Two distinct hypotheses have emerged from this observation. The first suggests that FLVCR1a plays a function that is fundamental only during the initial phases of erythroid differentiation, becoming dispensable in the later stages of maturation. According to this hypothesis, the decrease in FLVCR1a expression during erythroid differentiation appears as a passive consequence of progenitor maturation. The second hypothesis proposes that cells actively decrease FLVCR1a expression to facilitate maturation because this step is essential for triggering/release some crucial events required for cell differentiation. In this perspective, FLVCR1a becomes an active participant in the transition from a proliferative/undifferentiated state to a differentiated one.

According to the first hypothesis, it was postulated that FLVCR1a provides a mechanism to prevent high levels of heme in erythroblasts, which are highly sensitized to its toxic effects, and that FLVCR1a function becomes dispensable as erythroid cells mature, being heme incorporated into hemoglobin [4]. The consideration of FLVCR1a as a crucial molecule involved in the prevention of heme toxicity was also supported by our research, as we documented the significance of this protein in averting heme-induced oxidative stress and lipid peroxidation across various experimental models [7–9, 28]. This interpretation was perfectly in line with a role of FLVCR1a as a heme exporter, which acts as a detoxifying system to extrude the excess of intracellular heme. However, also choline import by FLVCR1a might be advantageous to mitigate heme toxicity, although the underlying rationale for this effect may not be immediately apparent. One possibility might be that choline is protective due to the relationship between choline metabolism and glutathione production [45–48]. Alternatively, choline may somehow affect intracellular heme levels, thus limiting heme toxicity by an indirect mechanism.

**Fig. 2:**
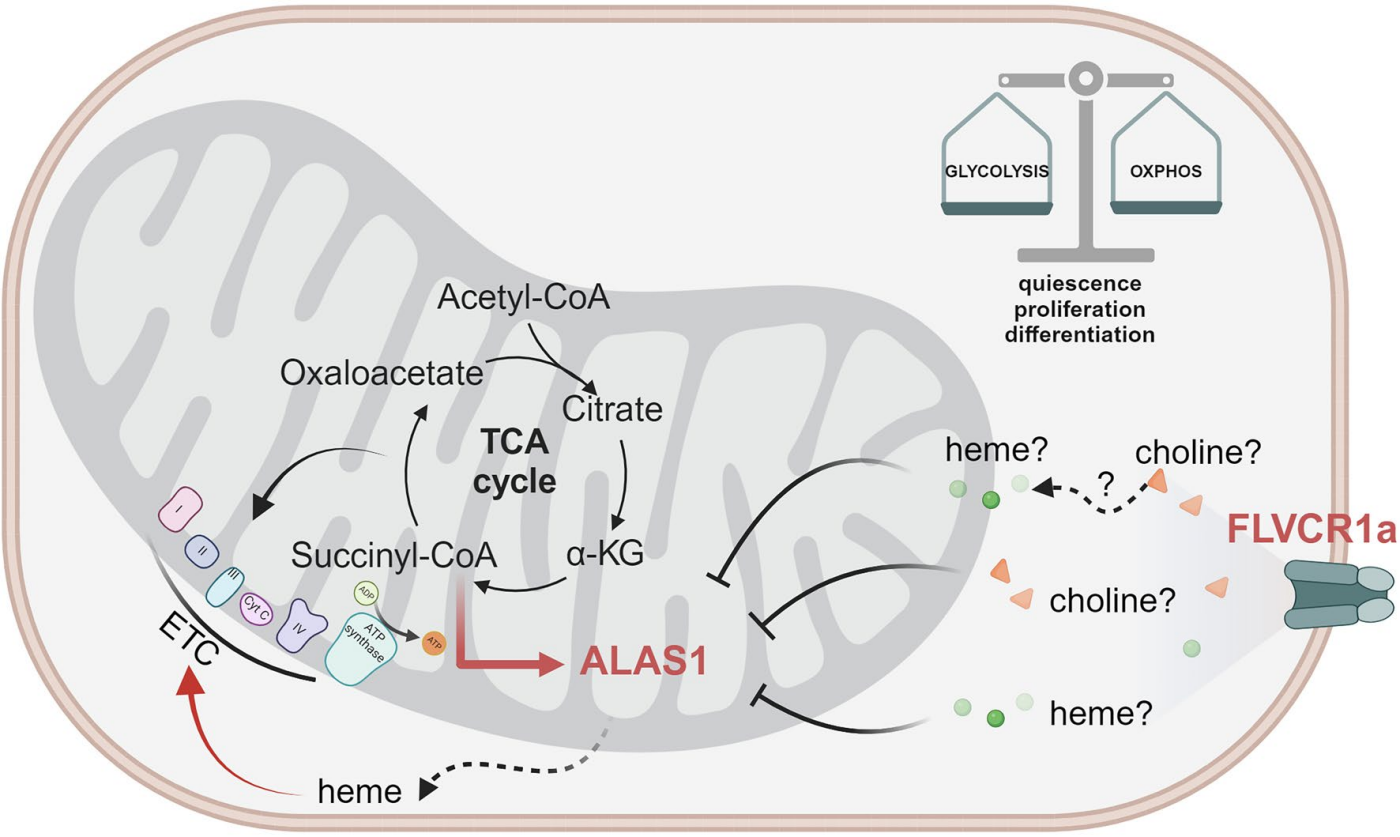
The FLVCR1a-ALAS1 axis plays a role in the metabolic rewiring that cells undergo as they transit between quiescence, proliferation, and differentiation states. By modulating ALAS1 either through choline or by directly/indirectly controlling heme levels, FLVCR1a impacts the cataplerotic function of ALAS1 on the tricarboxylic acid (TCA) cycle and its capacity to supply heme to the electron transport chain (ETC). This, in turn, influences the metabolic preferences of cells, leading them towards a more glycolytic or oxidative metabolism. These metabolic shifts influence the cells' ability to maintain quiescence, support proliferation, or undergo differentiation. The figure was created with BioRender.com

Nevertheless, in addition to FLVCR1a role in limiting heme toxicity, the involvement of this transporter in cell differentiation can also be explained from another perspective. Our laboratory has observed a significantly high expression of *Flvcr1a* during the initial stages of embryonic development (specifically at E9.5), followed by a gradual decrease in expression as development progresses (namely at E11.5, E13.5 and E15.5) [39]. This pattern suggested us that elevated FLVCR1a levels were a common characteristic of actively dividing and undifferentiated cells. Conversely, as cells underwent differentiation, *Flvcr1a* expression diminished. Consequently, the regulation of FLVCR1a expression during cellular differentiation appeared to extend beyond the erythropoietic compartment, in tissues where potential risks associated with excessive heme production were presumably minimal. These observations prompted us to contemplate the possibility that FLVCR1a's role in differentiation went beyond the previously described detoxification function. The discovery of a functional relationship between FLVCR1a and the heme biosynthetic enzyme ALAS1, which plays a crucial role in regulating the metabolic state of cells [27], offered us valuable new insights. Indeed, we demonstrated that FLVCR1a function was strictly associated with ALAS1-mediated heme synthesis, so that reduction in FLVCR1a function usually correlated with decreased heme production. Moreover, we showed that, by modulating heme biosynthesis, FLVCR1a finely balanced the two opposing and complementary effects exerted by ALAS1 on oxidative metabolism. Indeed, on one hand, ALAS1 supports oxidative phosphorylation (OXPHOS) by supplying the heme cofactor to the complexes of the electron transport chain (ETC). On the other hand, it opposes OXPHOS by consuming succinyl-CoA, serving as a cataplerotic pathway of the tricarboxylic acid (TCA) cycle, which limits its ability to provide reducing equivalents to the ETC. Therefore, we concluded that, as part of a common functional axis linked to the TCA cycle and OXPHOS, FLVCR1a and ALAS1 contribute to determine the cellular transition between the glycolytic metabolism and OXPHOS. This model suggested us the possibility that cells leverage the adjustment of FLVCR1a expression to reconfigure their energy metabolism, a vital necessity during the various stages of differentiation and the shift from proliferation to differentiation (Fig. 2).

Several pieces of evidence support this hypothesis. Indeed, FLVCR1a expression decreases during erythroid cell differentiation [1] in concomitance with reduction of glycolysis and transition to an oxidative metabolism [49]. Moreover, tumor cells exhibit elevated FLVCR1a expression to ensure the maintenance of their proliferation state [11, 27, 37, 38, 50], whereas depletion of FLVCR1a counteract tumor growth and shift tumor cell metabolism from a glycolytic to a more oxidative one [27]. Likewise, this is also in line with the reduced intestinal mucosa regeneration upon ulcerative colitis observed in intestinal specific *Flvcr1a*-null mice [9]. In addition, this model also give reason to the HIF-mediated upregulation of FLVCR1a expression in response to reduced oxygen availability [41].

Furthermore, this interpretation well aligns with the peculiar relevance of FLVCR1a for vascular development, a compartment highly dependent on glycolysis. Indeed, beyond the hematopoietic defects, the primary phenotype emerging from both *Flvcr1a*^−/−^ mouse embryos and *Flvcr1a*-downmodulated zebrafish morphants generated in our lab was the severe compromission of vasculature. In zebrafish embryos injected with two distinct morpholinos targeting *Flvcr1a* mRNA, the structural integrity of intersegmental vessels was completely disrupted at 48 h post-fertilization, and the injection of *Flvcr1a* cRNA into these zebrafish morphants resulted in the full restoration of angiogenic functionality [39], indicating a role of the transporter in the formation of a functional vascular network during embryogenesis. In *Flvcr1a*^−/−^ mouse embryos we observed reduced vasculature extension and complexity compared with controls, as well as multifocal and extended hemorrhages, evident in the primordial limbs at E12.5 and then throughout the head and body, associated with subcutaneous edema [18]. These observations were confirmed in constitutive (*Flvcr1a*^flox/flox^;Tie2-Cre mice) [8] and inducible (*Flvcr1a*^flox/flox^;Cdh5-Cre^ERT2^) [39] endothelial specific *Flvcr1a*-null mice. Indeed, constitutive disruption of *Flvcr1a* in endothelial cells of *Flvcr1a*^flox/flox^;Tie2-Cre mice resulted in embryonic lethality between E14.5 and E16.5, with massive intraembryonic bleeding visible in the distal regions of the body starting from E12.5, associated to edema and skeletal malformation in limbs, but there were no apparent gross morphological alterations in major organs. The structure of the vascular network was severely impaired in these embryos, with altered and enlarged microvessels [39], and their endothelial cells exhibited intracellular vacuoles often originated from an enlargement of the endoplasmic reticulum and mitochondria, morphological features typically observed in cells undergoing paraptosis, a specific type of programmed cell death [8]. Conversely, postnatal mice lacking *Flvcr1a* in the endothelium (*Flvcr1a*^flox/flox^;Cdh5-Cre^ERT2^) did not show apparent vascular defects, except in those tissues undergoing postnatal vascular remodeling, as the retina, or when vascular remodeling was stimulated by pathological conditions, as the induction of tumors [39]. These findings led to the conclusion that FLVCR1a plays a crucial role in physiological angiogenesis, as well as in the development of pathological tumor-associated angiogenesis, both of which involve the formation of blood vessels through active proliferation of endothelial cells sustained by glycolytic metabolism. On the other hand, it appears that FLVCR1a becomes less necessary for the maintenance of quiescent endothelial cells in adult vascular beds, particularly when the vascular network has already reached a remodeled and stable structure [39].

Finally, a definitive validation of the significance of the FLVCR1a-ALAS1 axis in shaping the metabolic conditions necessary for cells to transition from proliferation to differentiation came from our recent study on skeletal muscle specific *Flvcr1a*-null mice [10]. The diverse metabolic profiles displayed by skeletal muscle fibers, required to match energy needs with contractile demands, enabled us to analyze the effects of FLVCR1a deficiency across various cellular metabolic contexts simultaneously. In skeletal muscles, we validated that FLVCR1a deficiency leads to decreased ALAS1 activity. Additionally, we observed that *Flvcr1a*-null muscles, when injected with cardiotoxin (CTX) in tibialis anterior to induce muscle damage, exhibited delayed regenerative responses. This delay was linked to the inability of CTX-injured *Flvcr1a*-null muscles to elevate glycolysis during regeneration, resulting in a precocious shift towards oxidative metabolism. In vitro studies showed that satellite cells isolated from *Flvcr1a*-null muscles spontaneously formed larger myotubes when maintained in proliferating medium, indicating a premature progression towards myogenic lineage. Similarly, *Flvcr1a*-null satellite cells displayed a significantly lower proportion of desmin-positive myotubes upon induction of differentiation, although they exhibited enhanced terminal myogenic differentiation features compared to control cells. This suggests that FLVCR1a deficiency leads to premature myogenic differentiation, possibly by triggering an early transition from glycolysis to OXPHOS. Consistent with this, we found significantly lower *Flvcr1a* expression in activated (differentiating) compared to inactivated (proliferating) wild-type muscle satellite cells, as well as in C2C12 myogenic cells cultured in differentiation medium compared to those in proliferating medium. Importantly, we demonstrated that the premature differentiation in *Flvcr1a*-null satellite cells was dependent on reduced ALAS1 activity, as treatment of satellite cells with agents affecting heme biosynthesis mirrors/reverses the abnormal myogenic commitment seen in *Flvcr1a*-null satellite cells. Our findings align with recent research implicating heme biosynthesis in the naïve-to-primed embryonic stem cells transition [51], a shift from two different pluripotent states exhibiting contrasting metabolic requirements [52].

Drawing from this evidence, we posit that FLVCR1a's role in modulating energy metabolism better elucidates its involvement in cell differentiation as compared to the detoxifying function. Importantly, it's crucial to emphasize that this proposed model aligns with FLVCR1a's functionality, serving either as a heme exporter, a choline importer, or a versatile dual-purpose transporter. Indeed, the connection between FLVCR1a and ALAS1-mediated heme synthesis is indisputable, regardless of the substances transported by FLVCR1a and regardless of whether FLVCR1a's impact on cellular heme metabolism is direct or indirect.

## FLVCR1a in pathological scenarios

In the previous paragraphs we managed to summarize the studies, collected over the past two decades, that contributed to elucidate at least in part the features and the physiological role exerted by FLVCR1a. However, from the moment of its discovery, it became immediately evident that the interference of FLVCR1a function led to severe outcomes. Indeed, the interaction of this receptor with the FeLV-C virus resulted in red cell aplasia, and the inhibition of FLVCR1a activity within the human erythroid cell line K562 hindered the maturation of erythroid cells and triggered apoptosis [1], signifying serious repercussions on erythroid cells when FLVCR1a experiences a disruption in its function. Moreover, beyond the effects on the erythroid compartment, the complete compromission of FLVCR1a during embryonic development was shown to be incompatible with sustaining life, and postnatal depletion or malfunction of FLVCR1a was demonstrated to impair cell functions, with varying degrees of severity depending on the affected tissue. Therefore, the following paragraph will provide an overview (i) of the pathological consequences resulting from defective FLVCR1a function and (ii) of the most significant pathological conditions that seem to exploit dysregulation of FLVCR1a for their initiation or maintenance.

### Pathological consequences resulting from defective FLVCR1a function

The observations in mouse and zebrafish models with disrupted FLVCR1a, along with the findings in cells infected by the FeLV-C virus and in *FLVCR1a*-silenced human cell lines, all point towards the primary importance of FLVCR1a in the erythroid and vascular systems. Nevertheless, while the animal models developed proved highly valuable in uncovering the physiological role played by FLVCR1a in the metabolic rewiring crucial for cell differentiation, it is essential to acknowledge that they did not provide comprehensive insights into the specific mechanisms underlying human pathologies resulting from defective FLVCR1a.

Initially, the phenotypes observed in *Flvcr1*-deficient animals prompted extensive efforts to establish a causal link between FLVCR1a dysfunction and hematological disorders. The arrest of CFU-E/proerythroblast, marrow morphology, and clinical findings observed in FeLV-C viremic cats closely resembled those found in Diamond-Blackfan anemia (DBA) and myelodysplastic syndrome (MDS) associated with isolated del(5q) patients [53]. Furthermore, the absence of definitive erythropoiesis, coupled with craniofacial and limb anomalies observed in *Flvcr1*-deficient animal models, mirrored recurrent phenotypes in DBA patients. Therefore, the research focused on DBA. Studies conducted successfully demonstrated enhanced alternative splicing of *FLVCR1*, leading to the production of non-functional proteins, in CD71 (high) cells derived from human DBA patients [54]. Additionally, dysregulated expression of both *FLVCR1a* and *FLVCR1b* isoforms was observed in a cellular model of DBA [55]. Recently, two patients closing mirroring the *FLVCR1-*null phenotype in humans were identified. These patients harbor a homozygous 4-bp deletion in the *FLVCR1* gene, which causes a splicing defect (skipping of exon 9) and an early truncation of the protein. Noteworthy, they exhibit microcephaly, intrauterine growth retardation, severe craniofacial dysmorphism with clefting, and significant skeletal malformations, in a pattern highly consistent with Diamond-Blackfan syndrome [56]. Nevertheless, despite these findings, growing evidence indicate that the phenotype of human individuals with mutations in *FLVCR1* is significantly more heterogeneous and demonstrates a higher level of complexity than what was observed in FLVCR1a-deficient animals.

Indeed, a homozygous protein truncating variant in *FLVCR1* gene was identified in a patient with a clinical diagnosis of Walker Warburg Syndrome (WWS), a severe form of dystroglycanopathy-type congenital muscular dystrophy [57]. The patient presented with early onset hypotonia, profound hydrocephaly, myopathic changes on electromyography, contractures, seizures and optic nerve atrophy. Furthermore, most patients harboring mutations in the *FLVCR1* gene have been reported to suffer from a childhood-onset autosomal-recessive disorder known as Posterior Column Ataxia and Retinitis Pigmentosa (PCARP) [58–63], by non-syndromic Retinitis Pigmentosa (ns-RP) [64–68], and by Hereditary Sensory and Autonomic Neuropathy (HSAN) [28, 32, 69–71]. These disorders exhibit partial overlap and share common features, including the gradual degeneration of photoreceptors and/or sensory neurons. This degeneration leads to progressive vision loss, sensory ataxia, and, in some cases, a lack of pain sensitivity. Additionally, variable features of these FLVCR1-related diseases encompass conditions such as scoliosis, camptodactyly, achalasia, gastrointestinal dysmotility [61, 72], as well as tremors [73], learning disabilities, and developmental delays [74]. Remarkably, erythropoiesis is not compromised in these patients.

The majority of PCARP patients exhibit homozygous missense mutations in the *FLVCR1* gene, impacting highly conserved residues critical for the expression, localization, and proper folding of the FLVCR1 protein. Conversely, in the case of HSANs patients with *FLVCR1* mutations, the situation is more heterogenous: two patients, initially reported in 2016 [28] harbor a missense mutation on one allele and a severe frameshift mutation on the other allele. The latter is likely to produce a truncated FLVCR1a protein that may undergo rapid degradation [28]. Moreover, a third patient, identified in 2017, carries a missense homozygous mutation [32]. In contrast, two patients documented in 2019 [71] show biallelic compound mutations that affect the translation initiation codon. In all these FLVCR1-related HSANs cases, the predominant characteristic is the absence of sensations related to pain and temperature. This often results in chronic ulcerations on the hands and feet of affected patients. These lesions can result in severe complications, such as soft tissue infections and osteomyelitis, and may lead to the amputation of the affected limb. In fibroblasts and lymphoblastoid cell lines derived from these patients we detected dysregulated heme metabolism, that is exacerbated by enhancing heme biosynthesis in these cells [28, 32, 71]. These discoveries align with FLVCR1a function as a heme exporter or, alternatively, in consideration of the suggested function of FLVCR1a in choline import, could also stem from an indirect effect on heme triggered by impaired choline metabolism.

What seems to be evident is that these mutations do not entirely disable the transporter's functionality. This observation, on one hand, could account for the discrepancy with the mouse models entirely deficient for FLVCR1a, and on the other hand, it opens up a potential therapeutic opportunity.

In line with this, based on their discoveries on the role of FLVCR1a in choline import and on the association of FLVCR1 gene variants with serum choline levels in humans, Birsoy’s group has proposed that patients with mutations in *FLVCR1* may benefit from dietary choline supplementation [12]. This proposal was supported by the observation that even a relatively modest supplementation of choline to pregnant mice was effective in partially rectifying some metabolic abnormalities observed in *Flvcr1*^*−*/−^ embryos, ultimately enhancing their viability until later embryonic stages [12]. In our opinion, at the present stage, the rationale for this approach is not completely supported by the data. Indeed, the effects of choline supplementation on *Flvcr1*^*−*/−^ embryos, despite encouraging, are extremely limited. Moreover, although Hamachi’s group and Nguyen’s group have revealed that disease-associated mutations correlate with a diminished choline-importing function of FLVCR1a [13, 14], additional studies conducted in physiologically relevant contexts, utilizing appropriate disease-related cell types, and employing in vivo models are necessary to validate the significance of these findings. Therefore, future studies are mandatory to help to rule out or sustain this possibility.

Furthermore, regardless of the specific cargo transported by FLVCR1a, we believe that it is essential to pinpoint the exact downstream process influenced by impaired transport activity, in order to unveil the root cause of these disorders and to formulate appropriate therapeutic interventions. Considering the physiological role of FLVCR1a in the metabolic rewiring necessary for cell differentiation, it is tempting to speculate that sensory neurons and photoreceptors could be the cell types most sensitive to dysregulation in the balance between OXPHOS and glycolysis for their proper differentiation, maturation, and proliferation.

### Pathogenic utilization of FLVCR1a dysregulation in disease initiation and persistence

**Fig. 3:**
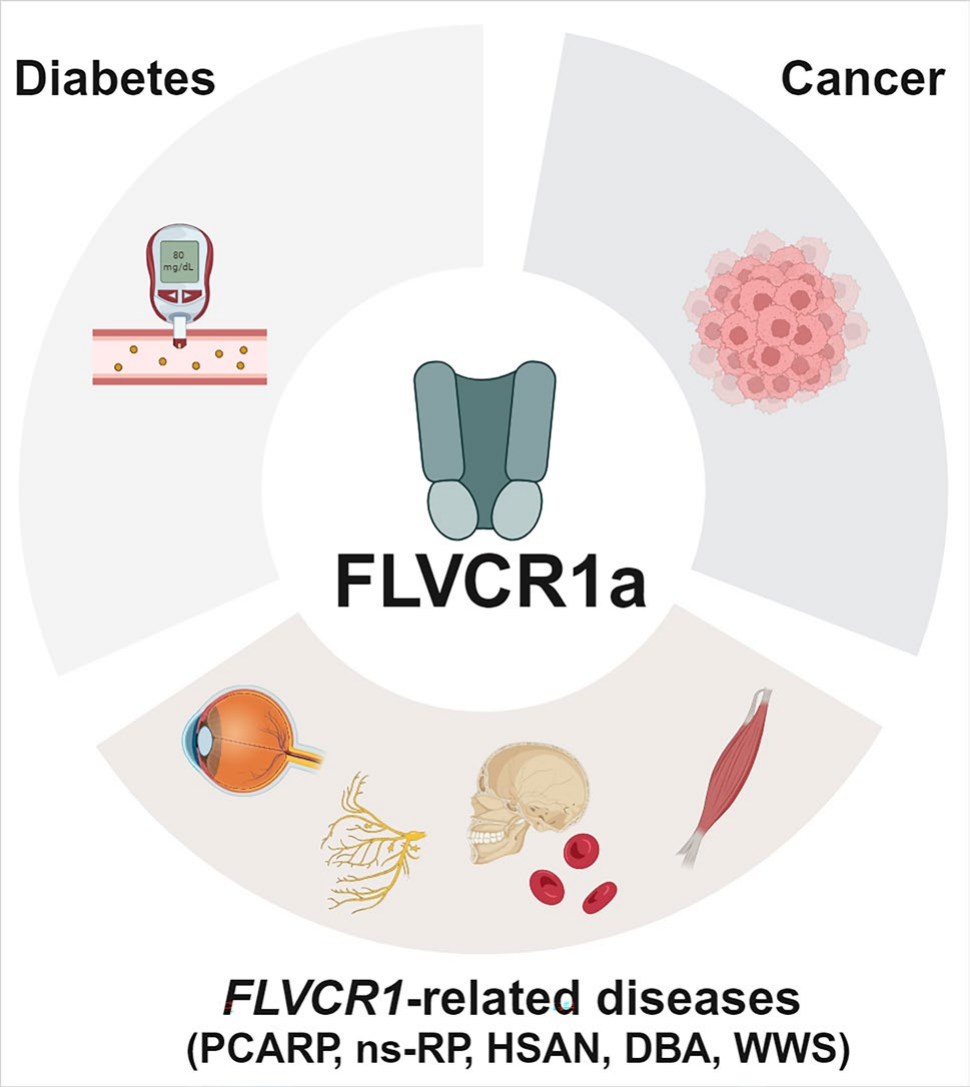
Pathological conditions arising from, or being sustained, by FLVCR1a mutation/dysregulation. The disruption of FLVCR1a function results in severe outcomes, manifesting as *FLVCR1*-related diseases such as Posterior Column Ataxia and Retinitis Pigmentosa (PCARP), non-syndromic Retinitis Pigmentosa (ns-RP), Hereditary Sensory and Autonomic Neuropathy (HSAN), Diamond-Blackfan anemia (DBA) and Walker Warburg Syndrome (WWS). Furthermore, aside from disorders arising from *FLVCR1* gene mutations, additional studies suggest that FLVCR1a dysregulation is associated with diabetes and cancer, potentially linked to its modulatory role on ALAS1. The figure was created with BioRender.com

Beyond the disorders arising from *FLVCR1* gene mutation, multiple studies indicate that FLVCR1a dysregulation is linked to additional pathological conditions, as diabetes and cancer and, especially for cancer, FLVCR1a dysregulation appears to be harnessed for disease maintenance and/or initiation (Fig. 3).

In 2017 Moreno-Navarrete et al. reported a significant increase of *FLVCR1* expression in both the visceral and the subcutaneous adipose tissue of patients affected by type 2 diabetes (T2D) as compared to non-diabetic subjects [75]. Moreover, in the same work the authors showed that *FLVCR1* expression was positively correlated with fasting glucose levels and other T2D-associated metabolic traits [75]. These data suggested that cells in the adipose tissue of T2D patients promote *FLVCR1* expression in response to impaired  systemic glucose metabolism.

Although these findings were limited to a single study, they appear particularly intriguing when considering the link between diabetes and ALAS1-catalysed heme synthesis. Indeed, the process of heme synthesis, especially within the liver, is intricately controlled by glucose. This regulation involves the suppression of ALAS1 expression by both glucose and insulin, and conversely, the promotion of ALAS1 transcription by fasting, thanks to a mechanism orchestrated by PGC-1alpha (peroxisome proliferator-activated receptor gamma coactivator 1alpha) [76]. Furthermore, it's worth noting that animals with a single functional copy of the ALAS1 gene (ALAS1 heterozygous mice) exhibit a prediabetic phenotype when maintained under normal feeding conditions, along with the development of age-dependent glucose intolerance and insulin resistance [77]. A plausible working model emerging from these observations involves the notion that under conditions of restricted glucose availability, ALAS1 activity is promoted to facilitate glucose uptake. Increased intracellular glucose levels, in turn, inhibit ALAS1 expression, and this repression halts the stimulation of glucose uptake.

Therefore, according to this model, and recognizing the functional association between FLVCR1a and ALAS1, the overexpression of *FLVCR1* in the cells of T2D patients may be interpreted as a cellular attempt aimed at heightening ALAS1 activity, in order to boost glucose uptake  in adipocytes, a process known to exert significant impacts on overall glucose regulation throughout the body, extending beyond its effects on adipocytes [78]. Hence, these insights strongly suggest that the association between FLVCR1a expression and diabetes reported in [75] could very well be attributed to FLVCR1a's impact on ALAS1 activity.

Nevertheless, currently, our understanding of the mechanism that could potentially link ALAS1 to the regulation of glucose uptake remains limited. Additionally, the restricted body of research exploring the connection between FLVCR1a and diabetes prevents us from reaching a conclusive comprehension of the transporter's role in this condition. Thus, it is imperative that additional investigations are conducted in the future to disentangle this complex issue.

The intricate connection between FLVCR1a, ALAS1 and glucose utilization is well exemplified in another pathological condition associated to aberrant FLVCR1a modulation, which is cancer.

Building on the discovery of diminished cell proliferation in the normal intestinal mucosa of an intestine-specific *Flvcr1a*-null mouse model [9], in 2021 our research group undertook an inquiry into the consequences of FLVCR1a depletion on tumor development [27]. Our study was preceded by a series of works reporting increased FLVCR1 expression in bovine papillomavirus-associated urinary bladder cancer [50], human synovial sarcoma [37], and human hepatocellular carcinoma [79]. In the case of hepatocellular carcinoma, elevated FLVCR1 expression was linked to more advanced disease staging, inflammation in adjacent tissues, vascular invasion, higher neoplasm histologic grade, and, notably, reduced overall survival and disease-free status [79]. In synovial sarcoma cells, when *FLVCR1* was silenced, it resulted in reduced cell proliferation, diminished cell survival, and decreased tumorigenicity both in vitro and in vivo [37]. Similarly, the silencing of *FLVCR1a* was shown to compromise the survival of neuroblastoma cells in vitro, particularly upon ALA administration [28].

Initially, our attention was directed towards examining the effect of FLVCR1a downmodulation on colorectal cancer growth [27]. We observed that *FLVCR1a*-silenced Caco2 proliferated more slowly than control cells did, and that *FLVCR1a* silencing in three different colorectal cancer cell lines, Caco2, SKCO1 and C80 cells, displayed a greater extent of basal apoptosis. In addition, subcutaneous injection of *FLVCR1a*-silenced SKCO1 cells in immunocompromised mice gave rise to smaller tumors than those obtained by the injection of control cells, confirming the inhibitory effect of *FLVCR1a* knockdown on CRC cell proliferation/survival in an in vivo context as well [27]. In our work, we were able to establish that enhanced expression of FLVCR1a in tumors plays a crucial role in supporting heme synthesis through ALAS1. Moreover, we were able to demonstrate that enhanced FLVCR1a-ALAS1 axis resulted in the downregulation of the TCA cycle, leading to decreased OXPHOS. Conversely, when we blocked this axis by inhibiting FLVCR1a or heme synthesis, the TCA cycle was fueled more vigorously by both glucose and glutamine, and OXPHOS was promoted. Based on these findings, we proposed that the heme synthesis-FLVCR1a axis contributes to metabolic adaptations that facilitate the growth of tumor cells by limiting oxidative metabolism in favor of aerobic glycolysis. This mechanism does not appear to be primarily dependent on heme itself. Indeed, it is not related to heme deficiency caused by ALAS1 downmodulation, as most of the effects we observed were replicated by cell treatment with ALA [27, 33], which increases intracellular heme content. Instead, the effects of the FLVCR1a-ALAS1 axis appears to be mostly based on the TCA cycle's cataplerotic action exerted by ALAS1. This system does not appear to be limited to colorectal cancer alone, as analyses on the BioXpress database [80] revealed that heightened FLVCR1a expression is a common characteristic across various types of cancer [27], and increased ALAS1-mediated heme biosynthesis is a recognized metabolic dependency in different kinds of tumors [11, 81, 82]. Furthermore, this also extends to tumor vasculature, as heightened FLVCR1a-ALAS1 axis, linked to increased glycolysis, is a common trait observed in tumor endothelial cells compared to their healthy counterparts [27, 83]. Notably, within tumor endothelial cells, the enhanced OXPHOS resulting from the downregulation of FLVCR1a on the one hand affects the state of the endothelial cells themselves, and on the other hand reshape the profile of metabolites derived from TCA-cycle related-processes, modifying the angiocrine properties of the endothelium, with an ensuing substantial influence on the tumor mass associated with the vasculature [83].

**Fig. 4:**
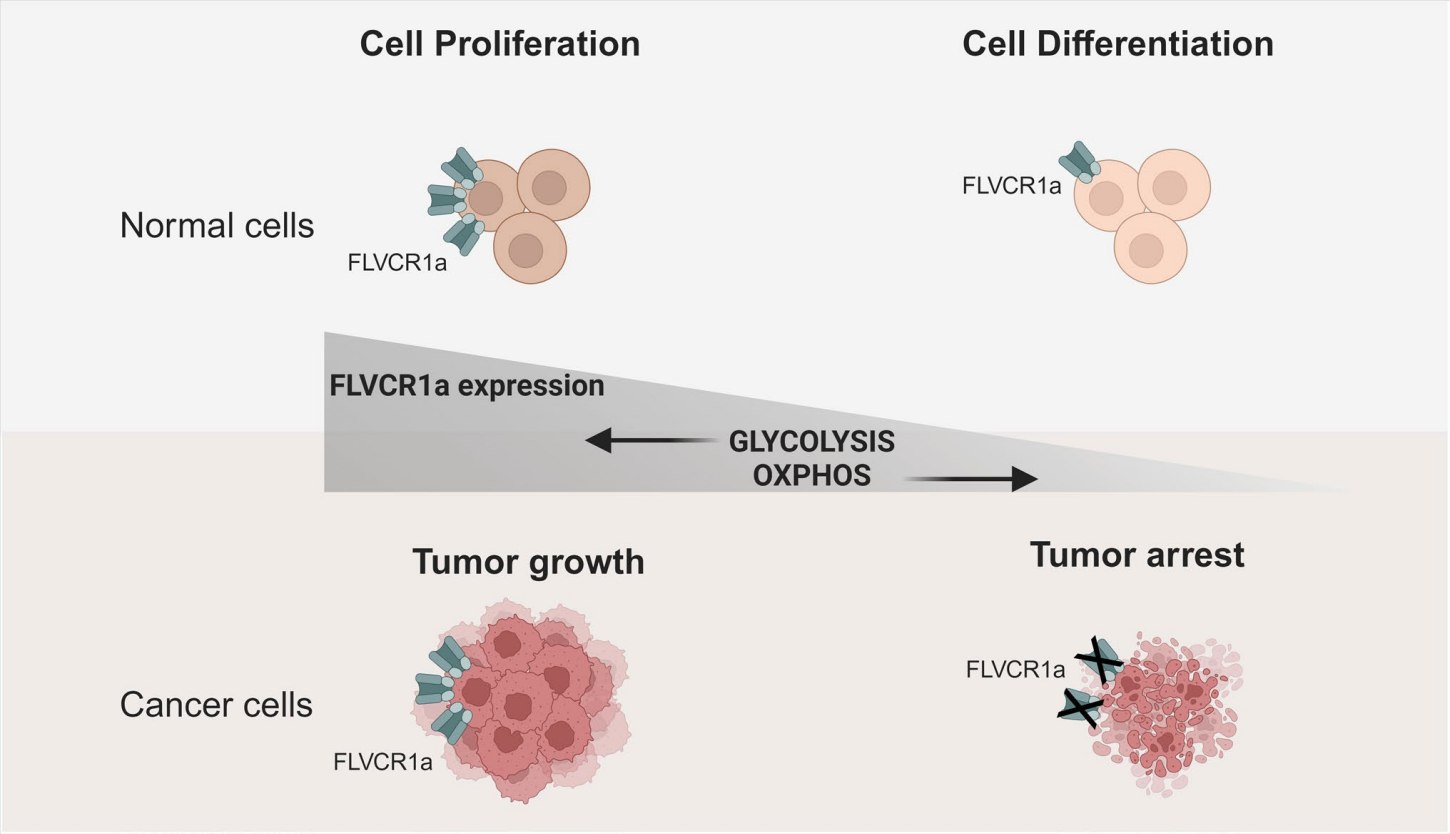
The expression of FLVCR1a impacts the metabolic profile of cells, leading to significant effects on cell fate under both normal and pathological conditions. In regular cells, reduced expression of FLVCR1a encourages a transition from glycolysis to OXPHOS, fostering cell differentiation. Conversely, in tumors, FLVCR1a is upregulated to maintain glycolytic metabolism. However, its induced reduction prompts a metabolic shift towards oxidative metabolism, which hinders tumor growth. The figure was created with BioRender.com

The suggested mechanism, indicating that tumor cells utilize the FLVCR1a-ALAS1 axis to restrict oxidative metabolism in favor of aerobic glycolysis, is in strong agreement with the putative role of ALAS1 in glucose regulation proposed above. Furthermore, this aligns with the upregulation of FLVCR1a expression in response to hypoxia [41], a condition that forces a metabolic shift away from OXPHOS. Similarly, it harmonizes well with the regulation of FLVCR1a expression during cell transition from an undifferentiated to a differentiated state, which is often associated with a change from glycolysis to OXPHOS [84]. Lastly, it is also consistent with the vital function of FLVCR1a in the normal development of the vascular system, as angiogenic endothelial cells primarily rely on aerobic glycolysis for their energy needs [85] (Fig. 4).

However, the recently proposed role of FLVCR1a as a choline importer introduces novel perspectives and implies that, in conjunction with the regulation of ALAS1 and heme biosynthesis, FLVCR1a's involvement in both diabetes and cancer may also hinge on choline-related mechanisms. We anticipate that forthcoming research will contribute to the elucidation of all these aspects and potentially facilitate their integration.

## Conclusions

In conclusion, roughly twenty years since its initial discovery, FLVCR1a continues to be an enigmatic transporter, with ongoing debates regarding its cargo and gene expression regulation. Nevertheless, despite this, the substantial body of evidence collected during these years has shed light on various crucial processes in which this transporter is intricately involved. This aspect adds an intriguing dimension to the forthcoming challenge of comprehending the specific substance transported and how this transport influences downstream processes. The future objectives are (1) elucidating whether FLVCR1a can indeed transport both heme and choline in opposite directions, (2) understanding the physiological significance of choline transport in comparison to heme transport and (3), if heme transport will be ruled out, determining how choline import by FLVCR1a can significantly impact heme metabolism, which is undoubtedly affected upon FLVCR1a dysregulation. This understanding is vital for unraveling the protein's physiological role and, consequently, for grasping the mechanisms underpinning the pathological consequences of its malfunction. In this context, future research questions will focus on understanding several key aspects: (1) the correlation between the extent of FLVCR1a malfunction and the type as well as the severity of pathological consequences; (2) the possible correlation between specific mutations in the *Flvcr1* gene and the predominantly affected tissue or process, and if so, the underlying reasons for this connection; (3) the distinction between pathological consequences related to FLVCR1a malfunction that are conserved across different species and those that are species-specific, and the underlying rationale behind this distinction; (4) the implementation of effective tools to improve or counteract FLVCR1a function and/or downstream pathways. Such knowledge will be pivotal for devising novel therapeutic strategies for pathological conditions linked to FLVCR1a to varying extents.

## Data Availability

Not applicable.
